# ILC2s—Trailblazers in the Host Response Against Intestinal Helminths

**DOI:** 10.3389/fimmu.2019.00623

**Published:** 2019-04-04

**Authors:** Tiffany Bouchery, Graham Le Gros, Nicola Harris

**Affiliations:** ^1^Department of Immunology and Pathology, Monash University, AMREP, Melbourne, VIC, Australia; ^2^Allergic & Parasitic Diseases Programme, Malaghan Institute of Medical Research, Wellington, New Zealand

**Keywords:** ILC2, helminth, type 2 immunity, activation, regulation, tuft cell, neuron

## Abstract

Group 2 innate lymphoid cells (ILC2s) were first discovered in experimental studies of intestinal helminth infection—and much of our current knowledge of ILC2 activation and function is based on the use of these models. It is perhaps not surprising therefore that these cells have also been found to play a key role in mediating protection against these large multicellular parasites. ILC2s have been intensively studied over the last decade, and are known to respond quickly and robustly to the presence of helminths—both by increasing in number and producing type 2 cytokines. These mediators function to activate and repair epithelial barriers, to recruit other innate cells such as eosinophils, and to help activate T helper 2 cells. More recent investigations have focused on the mechanisms by which the host senses helminth parasites to activate ILC2s. Such studies have identified novel stromal cell types as being involved in this process—including intestinal tuft cells and enteric neurons, which respond to the presence of helminths and activate ILC2s by producing IL-25 and Neuromedin, respectively. In the current review, we will outline the latest insights into ILC2 activation and discuss the requirement for—or redundancy of—ILC2s in providing protective immunity against intestinal helminth parasites.

## Introduction

Intestinal helminths have co-evolved with mammals and constitute a diverse but extremely successful group of pathogens infecting over one billion people worldwide, mostly in impoverished countries (1). Three main laboratory models of intestinal helminth infection have been used to study Group 2 Innate Lymphoid Cells (ILC2s). Two of these are rodent parasites (namely *Nippostrongylus brasiliensis* (Nb) and *Heligmosomoides polygyrus* (Hp)), that model human hookworm infection (2). Nb has a short life-cycle, that unlike its human counterpart causes only an acute infection, but nicely mimics the infectious lifecycle of the human hookworms by migrating from the skin to the lungs by the bloodstream, before being coughed up to finish its life cycle in the small intestine (2). Hp in the contrary, is a strictly enteric parasite, however like most human helminths this parasite establishes a chronic infection in its host and is strongly immuno-modulatory (2). The third model species, *Trichuris muris* (Tm) is used as a model of human whipworm infection, and is a non-migrating parasite that resides in the lumen of the large intestine (3).

Despite the diversity both in terms of biology of helminths, and their lifecycle within their host, the mammalian immune response against these parasites is remarkably conserved and is dominated by a type 2 cell mediated response, which is characterized by IgE antibody production, eosinophilia, mastocytosis, and the differentiation of type 2 macrophages (M2, activated either by IL-4 or IL-13) in response to the production of the canonical type 2 cytokines interleukin-4 (IL-4), IL-5, and IL-13 (4). More recently, the discovery of ILC2s, has forced us to re-evaluate the paradigm of what constitutes a protective immune response against these parasites.

ILC2s were first identified in 2010 as non T cell receptor (TCR) non B cell receptor (BCR) bearing cells that are enriched at mucosal sites of Nb infected mice (5). These innate cells were initially described as IL-25 responsive, and subsequently called ILC2 in a series of later publications that showed the importance of ILC2s for immune protection against helminths in primary infection (6–8). ILC2s are now understood to form part of a greater population of innate lymphoid cells, which also encompasses ILC1s and ILC3s, and are defined as lacking lineage markers (markers that define T cells, B cells, NK cells, myeloid cells, granulocytes, dendritic cells, and hematopoietic stem cells) in addition to expressing the transcription factors Gata-3 and (Retinoic Acid Receptor- Related Orphan Receptor Alpha) ROR-α (9). ILCs all originate from a common helper-like innate lymphoid precursor (CHILP) (10), whose development is regulated by Notch signaling and IL-7 (8, 11, 12). Id2, an inhibitor of E protein transcription factors, was been shown to be indispensable for ILC differentiation (6, 13). The factors driving the specific differentiation of ILC2s is still unclear, however it involves passage through an intermediate stage termed an ILC2-specific progenitor (ILC2P) (9). ILC2s express a variety of surface markers—most notably Chemoattractant Receptor-homologous molecule expressed on TH2 cells (CRTH2) (14), suppression of tumorigenicity 2 (ST-2), IL-17RB, CD127, CD80, MHCII and CD25, and produce the type 2 cytokines IL-13 and IL-5, as well as amphiregulin (8, 15).

Although early studies referred to ILC2s as a single population, these cells have more recently been described to exist as several subsets, termed natural (or tissue resident) ILC2s (nILC2s) and inflammatory ILC2s (iILC2s) (16). nILC2s are IL-33 responsive, express high levels of ST-2 and are not found in the circulation. By contrast iILC2s express high levels of Killer cell lectin-like receptor G1 (KLRG1) and IL-17RB, but low levels of ST2, and arise in response to IL-25 (16). Functional differences between iILC2s and nILC2s also exist, with iILC2s being described to produce more IL-13, whilst nILC2s exhibit a pro-repair phenotype and release IL-9 (16, 17). Plasticity between these subsets have been described with Notch ligands shown to promote the switch from nILC2 to iILC2 (12). Interestingly, the local microenvironment of the tissue or organ may influence the subset of ILC2s present. This hypothesis was recently confirmed by Huang and colleagues who reported that iILC2s expanded in the small intestine in response to helminth infection or exogenous IL-25 delivered intraperitoneally, but that intranasal administration of exogenous IL-25 did not induce iILC2s in the lungs (18). The authors hypothesized that iILC2s precursors are present in the small intestine, but not the lungs (18). Nevertheless, those iILC2s generated in the intestine were able to migrate to other organs, including the lungs, as demonstrated by elegant parabiosis experiments by the same authors (18). ILC2s from various tissue have a unique signature at steady state, as shown by single cells transcriptomic of ILC2s from gut, lung, skin and bone marrow (19). Furthermore, that different ILC2 subsets, or precursor subsets, may reside in different organs is supported by a recent study which intranasal administration of a unadjuvanted Fowlpox virus (FPV)-HIV vaccine caused nILC2s expansion locally within the lungs, whilst intramuscular administration of the same vaccine caused an expansion of iILC2s (20). Given that the existence of distinct ILC2 subsets is a relatively new finding we still have much to learn about its functional relevance and the majority of the literature discussed in this review reports work investigating ILC2s as a whole population rather than as distinct subpopulations.

Whilst the expansion and activation of ILC2s is likely to be beneficial for those living in regions endemic for helminths, their activity is more commonly associated with immune pathologies for those living in developed countries. These pathologies include airway hyperreactivity (21), allergen-induced lung inflammation (22, 23), and atopic dermatitis (24). Thus, an improved understanding of ILC2 activation and regulation is of great importance for human health. As a consequence, progress in this field demands that we understand the mechanisms involved in the activation and regulation of ILC2s as well as elucidating their full function. This review will focus on outlining the known role of helminth infection in promoting ILC2 activation, in addition to discussing their known functions during helminth infection. Specific attention will be given to the array of recent advances that have been reported using helminth models to study ILC2 function. Lastly, we outline what we believe are the most pressing questions for future research in this area.

## The Activation and Regulation of ILC2s

**Figure 1:**
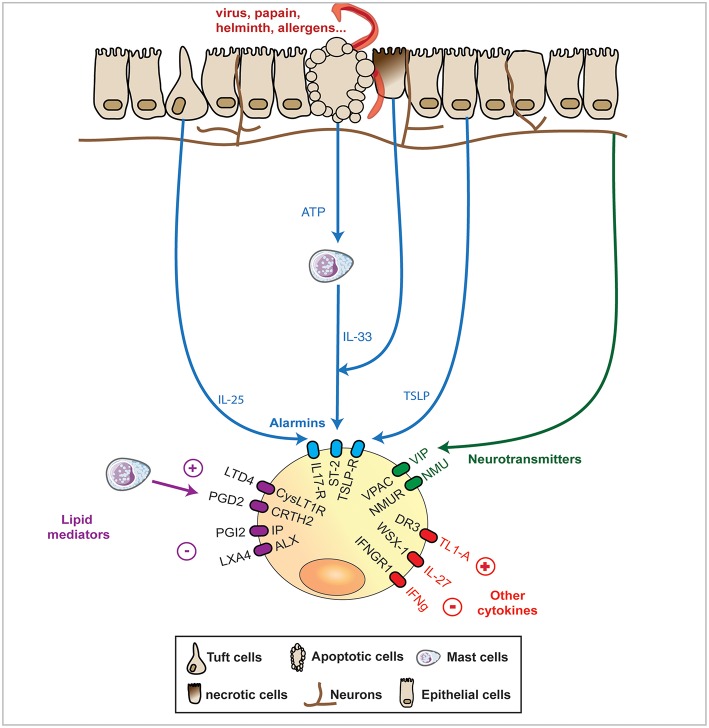
In response to various stimuli such as infection or injury, mucosal epithelial barriers express a range of signals that ILC2s can integrate. The most studied and thought to be of utmost importance pathway of activation of ILC2 is formed by the “alarmins,” IL-33, TSLP, and IL-25 that cause release of the canonical IL-13, IL-5, and amphiregulin cytokines expression. Upon damage, necrotic epithelial cells can release IL-33 while apoptotic epithelial cells, can release ATP, that further activates mast cells to release IL-33. Alternatively, epithelial cells can release TSLP or the newly identified and specialized chemosensory epithelial cells, namely tuft cells, can release IL-25. Recently mucosal sensory nervous system has been shown to detect helminths and protists and release in response Neuromedin (Nmu) or vasoactive intestinal polypeptide (VIP) that can both activates by themselves ILC2s or synergise with alarmins to potentiate ILC2 response. Finally, some cytokines and lipid mediators have emerged as potential controllers of ILC2s, with the prostaglandin PGI2 and the lipoxin LXA4 limiting ILC2 cytokines release, while the prostaglandin PGD2 and the leukotriene LTD4 can potentiate the same cytokine expression. The regulatory IL-27 cytokines as well as Interferon (IFN)g can dampen ILC2 activation.

Early and recent studies described the activation of ILC2s by a surprising array of stimuli including helminths, allergens, certain bacteria and even endogenous host molecules (8, 25–27). This is perhaps not surprising as it is now clear that ILC2s become activated in response to factors released by both stromal and immune cell populations in response to stress or tissue damage (as outlined below and in Figure 1). In the following paragraphs, we will discuss those pathways associated with the activation or regulation of ILC2 function to date.

### Epithelial Cell Production of Alarmins

Alarmins, namely Interleukin-33 (IL-33), IL-25, and Thymic stromal lymphopoietin (TSLP), can be released from a variety of cells, but are especially rich within epithelial cells present in the skin and mucosal tissues (28–32). These cytokines cause both the proliferation and activation of ILC2s and are particularly good at eliciting the production of IL-13 and IL-5 (33), with some reports also detailing release of IL-4 or IL-9 in mice (17, 27, 33, 34). The relative importance of each alarmin in ILC2 activation has been extensively studied, but still forms an incomplete picture. Studies suggest that IL-33 is more potent that TSLP or IL-25 at inducing ILC2s in the context of allergic airway inflammation (35). However, in the context of atopic dermatitis both TSLP and IL-33 have been shown to play a crucial role in ILC2 activation and pathology development, with TSLP specifically controlling the itch response (29) and IL-33 being more important for causing the “atopic march” (typical progression of allergic disease going from atopic dermatitis, to food allergy, rhinitis, and asthma) (36). Lastly, redundancy between all three cytokines has been observed in the context of fibrosis and chronic intestinal inflammation (37). Interestingly, TSLP and IL-33 can act in a synergistic manner during the host response to chitin stimulation, with the combination of alarmins acting to potentiate type 2 cytokine production by ILC2 (35). Such synergy was also observed for human ILC2s cultured *in vitro*, where the presence of two or three alarmins together promoted ILC2 proliferation and survival, and cytokine production (38). The presence of TSLP as a member of the “alarmin cocktail” was deemed to be of particular importance (38). Although the ability of all three alarmins to participate in ILC2 activation may at first be confusing, it has been proposed that the relative importance of each alarmin is related to the specific tissue, infection or pathology in which the ILC2 response is involved (39). This view suggests that the three alarmins do not act in a purely redundant, or even synergistic manner, but that the relative levels of each alarmin found within a given tissue may act to match the ILC2 response to environment in which it is present. In line with this view, IL-33 has been reported to mediate the activation nILC2s, whilst IL-25 preferentially elicits iILC2 activation (16). However, to date the full implications of these findings in terms of protective immunity or immune-pathology remain unclear.

All three alarmins are released by epithelial cells, although to varying amounts within distinct mucosal tissues. TSLP and IL-33 can be released by alveolar epithelial cells type II in the respiratory tract, by keratinocytes in the skin or by epithelial cells in the intestine. IL-33 is expressed by fibroblast reticular cells (28, 40) in lymphoid organs and myofibroblasts in the intestine (33). Endothelial cells in the spleen and lymph-nodes, as well as in the intestine under inflammatory conditions have been shown to be another source of IL-33 (28, 40). In human, it has recently been described that endothelial cells, rather than epithelial cells in the lungs release IL-33 (41, 42).

By contrast IL-25 production appears to be restricted to a specialized chemosensory epithelial cells, called tuft cells (30, 32, 43). These cells, which are also commonly referred to as “brush” cells, are found in the epithelium of various organs including the intestine and respiratory tract (44). In the intestine, tuft-cell-derived IL-25 elicits IL-13 release by ILC2s, which in turn promotes the further expansion of IL-25 producing tuft cells, in a feed-forward amplification of the type 2 immune response (32). Of note, expansion of intestinal tuft cells in response to Tm and Nb infection has been shown to be dependent on chemosensory taste receptors, most notably on the transient receptor potential cation channel, subfamily M, member 5 (Trpm5) (30, 45) which causes release of acetylcholine and activation of nearby vagal nerve fibers (46). Employing a single cell RNA sequencing approach, Haber and colleagues recently showed that intestinal tuft cells constitute a heterogeneous population, with two main subtypes identified (47). Both subsets expressed IL-25, but only one also expressed TSLP (and was also CD45+). Interestingly, the TSLP+ tuft cell population was specifically expanded following Hp infection (47). These data raise the question as to whether tuft cells respond differently to distinct stimuli and whether they can also promote ILC2 activation via TSLP. In the respiratory tract brush cells were shown to be activated by leukotrienes in response to *Alternaria* to release IL-25, which in turn activated ILC2s (48, 49). Unlike what has been described for intestinal tuft cells (30), respiratory brush cell hyperplasia was found to be STAT6 independent (48). Whether this difference is due to the tissue location of the cells, or to the source of the stimuli remains unclear.

The tuft cell/ILC2 expansion loop in helminth infection is now well established, but what triggers helminth recognition by tuft cells remains unclear. Recent work elegantly demonstrated that protists can be detected by tuft cells by virtue of their secretion of the metabolic product succinate (50). Surprisingly, however, even though Nb was shown to produce succinate, the tuft response to Nb infection was succinate independent, indicating the presence of an alternative stimulatory signal from this parasite (32, 50, 51). Another open question is whether negative regulators of the tuft cell-ILC2 feed-forward loop exist. In this regard, it was recently described that A20 (*Tnfaip3*) expression by ILC2s is a negative regulator of their expansion in response to IL-25 release by tuft cells in the intestine (50). Of interest, mice deficient for A20 within ILC2s exhibited intestinal crypt hypertrophy, thickening of the surrounding muscularis and an increased frequency of secretory cells, which are all features observed following helminth infection of wildtype mice (50).

### Nervous System

Sensory neurons have recently been shown by several groups (10, 52, 53) to contribute to the activation of an ILC2 response. The possibility that neurons may play a role in ILC2 activation was first raised by the finding that ILC2s expressed high levels of the Neuromedin U receptor 1 (Nmur1) (52). Nmur1 can be stimulated by its ligand, Neuromedin U (Nmu), which is typically expressed by cholinergic enteric neurons. Moreover, ILC2s have been reported to form close associations with Nmu+ neurons in both the lungs and intestine (52). Stimulation of ILC2s with Neuromedin U caused their prompt proliferation as well as eliciting expression of the type 2 cytokines IL-5, IL-13, the growth factors amphiregulin and colony stimulating factor 2 (Csf2) (10, 52). Interestingly, the response of ILC2s to Nmu was found to be more rapid than that observed for IL-33 or IL-25, suggesting that the neuronal/immune pathway could be a precocious threat sensor, activated even before the onset of tissue damage. In keeping with this hypothesis enteric neurons were shown to directly release Nmu in response to helminth products (52). That neuronal-ILC2 interactions play a functional role in helminth immunity was demonstrated by the more rapid expulsion of adult worms in Nb infected animals administered exogenous recombinant Nmu. NmU administration was also found to potentiate the response of ILC2s to IL-25, and to a lesser extent IL-33, as determined by IL-13 and IL-5 production *in vitro* (54). Synergy between Nmu and IL-25 was confirmed *in vivo* using an allergic airway inflammation model, with co-treatment increasing IL-13 and IL-5 level and eosinophils in the bronchoalveolar lavage (54). Furthermore, an *in vivo* synergistic role for IL-33 is also likely as Nmu deficient mice failed to exhibit increased numbers of ST2+ ILC2s (or nILC2s) in the lung of allergen challenged animals (54). Nmu release is not the only means by which neurons interact with ILC2s as a study investigating experimental allergic airway inflammation demonstrated a role for vasoactive intestinal peptide (VIP) in stimulating IL-5 release from ILC2s. VIP is produced by pulmonary neurons and can bind to the vasoactive intestinal peptide receptor 2 (VPAC2) present on ILC2s (53). Interestingly, IL-5 can in turn stimulate the release of VIP by sensory neurons, in yet another example of a feed-forward loop acting to amplify the type 2 immune response (53).

### Cytokines Other Than Alarmins

In both mouse and human, ILC2s express high levels of the tumor necrosis factor (TNF)-receptor superfamily member DR3 (TNFRSF25). Administration of recombinant TL1A, a DR3 ligand, induced ILC2 expansion and DR3 deficient mice are unable to expel Nb -highlighting this cytokine as a possible positive regulator of ILC2 function (55, 56). ILC2s can also respond to Transforming growth factor (TGF)-β, however the outcome of the response remains unclear. Epidermal-derived TGF-β has been shown to enhance ILC2 recruitment and IL-13 expression in the lungs after house dust mite exposure (57). By contrast TGF-β and IL-10 release by Tregs can suppress the production of type 2 cytokines by ILC2s *in vitro* and *in vivo* in an ovalbumin model of pulmonary allergy—although it did not impact on the proliferation and survival of these cells (58, 59). Given the contradictory nature of these reports more studies will be required to fully understand the impact of TGF-β on ILC2 function. Recently, skin ILC2s have been shown to be IL-18 responsive *in vitro*, causing IL-5 and IL-13 expression in these cells. This finding was further confirmed *in vivo* in an MC903 atopic like skin inflammation model, in which IL-18 deficient mice exhibit decreased ILC2s and eosinophils recruitment in the skin (19).

In terms of negative regulation, it is interesting to note that ILC2s can express the receptors for the anti-inflammatory cytokine IL-10, as well as for classical type 1 cytokines, such as Interferon (IFN)- γ and IL-27 (6, 60, 61). These cytokines are all able to suppress Th2 responses (62–64) and *in vitro* studies in which ILC2s were stimulated with IFN-γ, IFN-β, or IL-27 demonstrated that each of these cytokines could individually suppress the secretion of IL-13 and IL-5 by ILC2s (60, 65–68). A role for type 1 cytokines in restraining ILC2 function *in vivo* was demonstrated by adoptively transferring ILC2s isolated from the lung of wild type or IFNgr1^−/−^ Rag2^−/−^ mice into IL2rg^−/−^Rag2^−/−^ recipient mice (which lack endogenous ILC2 populations). Recipient mice were then given an intra-tracheal inoculation of recombinant IL-33 to activate the transferred ILC2s and the authors reported that ILC2s derived from IFNgr1^−/−^ Rag2^−/−^ mice produced higher quantities of type 2 cytokines compared to their WT counterparts (60). Mchedlidze and colleagues showed similar results using animals deficient in the IL-27 subunit Epstein-Barr virus induced gene 3 (Ebi3). *Ebi3*^−/−^ mice infected with Nb exhibited higher numbers of lung ILC2s and higher levels of circulating type 2 cytokines. The authors further confirmed that IL-27 regulation of ILC2 activation was direct by *in vitro* stimulation (68).

### Interaction With Innate Cells

Various innate cells have been shown to participate in the activation of ILC2s. IL-25 is known to be produced by eosinophils and mast cells (69), whilst IL-33 can be produced by mast cells (70, 71), and macrophages (72) indicating that these cells may be able to directly activate ILC2s. The activation of ILC2s can also result from interactions between innate cells and epithelial cells. IL-33—which is typically released as a procytokine—can be cleaved into its bioactive form by proteases released from mast cells and neutrophils (73, 74). This cleavage increases the potency of IL-33 by up to 10-fold, thus enhancing its ability to activate ILC2s (73, 74). In another example of epithelial cell-innate cell collaboration, intestinal epithelial cells release ATP that can activate mast cells, to secrete IL-33 and activate ILC2s (70, 75). More specifically, ATP activates mast cells by interacting with the adenosine receptor, P2X purinoceptor 7 (P2X7R) (70). Of note, ILC2s also express adenosine receptor and stimulation of bone marrow-derived ILC2s with the nonselective adenosine receptors agonist, 1-(6-samino-9*H*-purin-9-yl)-1-deoxy-*N*-ethyl-β-d-ribofuranuronamide (NECA), resulted in decreased type 2 cytokine production by these cells (70, 76). In response to Nb or Hp infection, blockade of the A2B adenosine receptor (A2BAR) inhibited ILC2 expansion and treated mice failed to expel the adult worms. However, whether the cells targeted by A2BAR blockade were ILC2s or epithelial cells was unclear (77). Altogether these studies highlight an interesting role of adenosine receptors in regulating ILC2 responses—but the exact molecular mechanisms by this occurs will require further study.

### Lipid Mediators

Arachidonic acid derivatives, including the cysteine leukotrienes LTC4 and LTE4, the prostaglandin PGE2, are well characterized for their role in the induction and control of type 2 inflammation (78). Not surprisingly, ILC2s have been shown to respond to various lipid mediators, which act either to activate or suppress the activity of these cells. Prostaglandin D2, a product of Prostaglandin D2 synthase, has been shown to promote ILC2 migration and IL-13 production both in humans and in mice (14, 79, 80). Indeed, the receptor for PGD2, CRTH2, has been highlighted as a useful marker of human ILC2s (81). Murine ILC2s express both CysLTR-1 and-2 and their ligands, LTC4, LTD4, and LTE4 have been shown to induce IL-13 and IL-5 expression by these cells (82, 83). In contrast, other eicosanoids, including lipoxin A4 (LXA4) and Prostaglandin I2 (PGI2) limited the activation of ILC2 and inhibited the production of type 2 cytokines (84, 85). Similar to the synergy noted between Nmu and alarmins, some leukotrienes (namely LTB4 and LT C4), have been shown to enhance the ability of IL-33 activate ILC2s in the context of lung inflammation (86) or helminth infection (83).

In summary, ILC2s are able to be activated by a large array of stimuli—a finding that, at least in part, explains the diversity of environmental triggers that can elicit a type 2 immune response. Further research as to how different stimuli co-operate to activate ILC2 and their possible relevance to different tissues and/or pathological settings will no doubt lead to the development of better therapeutics for type 2 mediated diseases. Last but not least it is likely that we have only exposed the “tip of the iceberg” in terms of identifying possible positive and negative regulators of ILC2 activation, and it is certain that this area will continue to yield exciting and novel insights into type 2 immune responses.

## ILC2 Function During Intestinal Helminth Infection

The extensive research dedicated to the activation and regulation of ILC2s (outlined above) has provided many answers related to the possible function of these cells and has also raised many questions. One key question is what is the relative role of these cells in mediating protective immunity and tissue repair in response to helminth infection. The following section will discuss the current state of the art in terms of our understanding of ILC2 function during intestinal helminth infection.

### A Role for ILC2s in Driving the Expulsion of Adult Worms

The expulsion of adult worms from the intestinal lumen has long been known to be associated with strong type 2 cytokine production, with IL-13 acting as a potent activator of epithelial cell turnover, goblet cell hyperplasia and mucus secretion, and increased muscle contractility—culminating in what is commonly referred to as a “weep and sweep” response (87). Although much of the early work on this response centered on the contribution of Th2 cells, we now understand that ILC2s are an important contributor of IL-13 produced early on during infection (88). Indeed, one of the founding papers reporting the existence of ILC2s, demonstrated that adoptive transfer of ILC2s into the normally susceptible IL-13 deficient mice was sufficient to promote the expulsion of Nb (8). This proved that although ILC2s are a rare cell type, they can actively contribute to anti-helminth immunity. ILC2s also produce IL-5 (and are considered as the main source for this cytokines in allergies) and can mediate an early Th cell-independent tissue eosinophilia (8). However, although eosinophilia is a hallmark of helminth infection, they have been described to exhibit diverse, even contrasting, roles in terms of protective immunity and thus their full function remains unclear (89).

To date, ILC2s have been shown to contribute to the timely expulsion of a variety of helminths including Nb and Tm (8, 90, 91). Amongst these parasites, Nb is the most potent elicitor of the ILC2 response, and both the recruitment and activation of ILC2s by IL-33 has been shown to be required for the expulsion of this parasite. Of note, whilst IL-25 is not required for the eventual expulsion of Nb (8), it is important for the expulsion of Hp (92). Intriguingly however, in Hp primary infection, enhanced ILC2 numbers caused by IL-2 treatment (up to 5 times their basal level) were not sufficient to cause adult worm expulsion (34). However, this treatment resulted in reduced adult worm burden with increased numbers of L4 larvae trapped in the submucosa (34). Similarly, treatment of Tm infected mice with recombinant IL-25 treatment promotes parasite expulsion, however this study was completed before the discovery of ILC2s (93). All in all, IL-25-induced ILC2 expansion and activation appears to play an important role in promoting the expulsion of adult helminth parasites, but the relative contribution, of these cells to host immune responses against distinct parasites remains unclear. Nevertheless, alarmin release and nervous recognition of helminths resulting in increased Nmu expression all seem to be a general feature in the host response to intestinal helminth infection (10, 94).

Although ILC2s have created much excitement, it is important to note that in natural settings the activation of ILC2s alone is not sufficient to mediate protection against helminths. Indeed, Neill and colleagues demonstrated that transfer of ILC2s into Rag2-deficient mice (which lack B and T cells) was not sufficient to mediate worm expulsion (8). In this setting, ILC2 numbers were not sustained for long enough (more than 2 days) to allow expulsion of the worms and the authors suggested that Th2 cells might support ILC2 maintenance (8). Indeed, a series of later reports identified an interplay between ILC2s and the adaptive immune response, and this will be discussed later in the review.

### The Contribution of ILC2s to Tissue Repair

Intestinal helminths are large multicellular pathogens that cause extensive tissue damage as they migrate through host tissues as larvae stages, and whilst they dwell within the intestine as adult worms. In recent years, is has become evident that type 2 immune responses evolved not only to limit parasite burdens, but also suppress excessive inflammation and to mediate the rapid repair of damaged tissues (95). To date, many of the investigations addressing the contribution for type 2 immunity to tissue repair have focused on IL-4 activated macrophages (95). However, studies addressing the possible contribution of ILC2s to repair are beginning to emerge. Amphiregulin (an epidermal growth factor) has long been known to be required for protective immunity following Tm infection (96), and ILC2s were later reported to represent a potent source of this cytokine (97). Studies investigating the role of IL-9 in helminth immunity noted that IL-9 deficient mice infected with Nb exhibited enhanced lung damage and delayed worm expulsion (17, 33, 98). The same authors demonstrated that IL-9 functioned as a survival factor for ILC2, which in turn provided the amphiregulin required for efficient lung repair following parasitic migration through this organ (17, 33). Similarly, nILC2s have been shown to secrete amphiregulin leading to the differentiation and proliferation of epidermal growth factor receptor (EGFR) expressing epithelial cells following respiratory virus infection (97). ILC2s may also promote intestinal protection against damage as the transfer of ILC2s in an experimental model of colitis was shown to attenuate disease severity, through enhanced mucin production (99). IL-9 production by ILC2s has also directly been shown to limit type 1 inflammation in a sepsis induced model of acute lung inflammation (27). In this study, it was further shown that IL-33 activated ILC2 present in the lung produced IL-9 which acted to prevent lung endothelial cells from undergoing pyroptosis (a form of cell death), by virtue of its ability to limit caspase-1 activation (27).

Together these studies indicate that ILC2s can contribute to the modulation of inflammation and the promotion of tissue repair following a variety of environmental insults. However, it is possible that these cells also contribute to the pathology that can result from exaggerated or prolonged type 2 inflammatory responses. On this note nILC2s have recently been shown to constitutively express arginase-1 (Arg-1), and the selective absence of this gene within ILC2s resulted in an exacerbated emphysema in response to Nb infection (100). Similarly, IL-13 secretion by ILC2s present in the lungs has been demonstrated to in the disrupt tight junctions in asthmatic patients (101).

### Adaptive Immune Response

#### Priming of Type 2 Immune Response

The cellular and molecular mechanisms that lead to the differentiation of naïve CD4+ T cells into type 2 cytokine producing T helper 2 (Th2) cells are still not fully understood. To date most of the work in this area has focused on the importance of dendritic cell (DC)—T cell interactions, however the discovery of ILC2s has widened our view of a DC centric world to appreciate the possible importance of ILC2s in initiating or modulating the Th2 response.

Studies using experimental mouse models in which ILC2s were preferentially depleted have revealed that ILC2s are required to promote Th2 cell responses in response to infection with Nb, or following the intranasal administration of the allergen papain (102–104). More recently, tissue-specific ILC2s were shown to represent a critical source of the co-stimulatory molecule OX40 ligand (OX40L) in response to IL-33 stimulation (105). Binding of OX40L to OX40 on CD4 T cells was required for the development of both Th2 and GATA3+/– Treg responses in the lungs after Nb infection (105). In keeping with these findings, ICOS-ICOSL interactions between ILC2s and CD4 T cells have been shown to be required for optimal Treg expansion in response to IL-33 stimulation or Nb infection (67). ILC2s can also contribute to the development of Th2 cells in response to the murine helminth Hp infection by releasing IL-4 (34, 103). Although most studies indicate that murine ILC2s make little IL-4, this is in contrast to human ILC2s which can produce large quantities of this cytokine in response to combined stimulation with IL-33 and TSLP (106).

In addition to direct ILC2-T cell interactions, ILC2s can impact on DC function and have been shown to promote the migration of DCs from the tissues to the lymph node by virtue of producing IL-13 (102). Last but not least, ILC2s can present antigen directly to CD4 T cells (103) and CD4 T cells have been shown to support the continued survival of ILC2s by providing IL-2 (107, 108). Altogether, these studies highlight a complex interplay between ILC2s, DC and CD4 T cells that promotes the development of optimal adaptive type two immune responses.

#### Memory Immune Responses

ILC2s have now been reported to contribute to the amplitude of memory type 2 immune responses in a variety of models. In the first report, Halim and colleagues investigated the contribution of ILC2s to recall responses against papain (109). Here, DCs play a critical role by producing CCL-17 and CCL-22 to attract CCR4+ memory CD4 T cells. Interestingly the expression of CCL-17 and CCL-22 was triggered by type 2 cytokines released by ILC2s - and ILC2 ablation prior to papain re-challenge attenuated the number of Th2 cells present (109). In another example of ILC2 potentiation of memory type 2 responses, these cells were reported to critically contribute to the production of IL-13 in response to challenge infections with Nb allowing the rapid activation of M2 macrophages which were able to mediate both parasite killing and tissue repair (108). In this model, Th2 cells also contributed to the activation of M2 macrophages by producing IL-4, and were additionally found to promote the maintenance of ILC2s following challenge infection with Nb in an elegant example of ILC2-Th2 cell co-operation (108). Of note, short term treatment with recombinant IL-33 has been reported to induce the sustained activation (for over 1 year) of ILC2s both in helminth and glomerulosclerosis models indicating that—like memory cells—some ILC2s could be long lived cells (110, 111). Lastly, previously activated ILC2s were able to produced increased amounts of IL-13 when re-exposed to the same antigen, or even to an unrelated allergen or to IL-33 (110).

#### Humoral Immune Responses

The role of ILC2s in humoral immunity has just begun to be addressed. Recently, ILC2s isolated from the lungs of naive wild type mice were shown to promote the proliferation of B1-, as well as B2-, type B cells *in vitro*. ILC2-activated B cells produced IgM, IgG1, IgA, and IgE, with the production of IgM being IL-5 dependent (112). Given that antibodies, and in particular IgE and IgG have been implicated in protective immune responses against challenge infections with a variety of helminths (113, 114), the impact of ILC2 on B cell responses could be of great importance to helminth protection. Moreover, one of the functions of IgE is to arm basophils and mast cells, that in turn function to potentiate Th2 responses (115), or release inflammatory mediators (116) potentiating type 2 inflammation.

### Helminth-Mediated Regulation of the ILC2 Response

Helminths have co-evolved with their host and typically form chronic infections in their host. These parasites are often described as masters of immunomodulation and a multitude of parasite-derived products have been identified that interfere with host immune responses (117). It is noteworthy that Nb, which is a potent elicitor of ILC2 responses, is expelled rapidly from its murine host, whilst Hp and Tm, which elicit only modest ILC2 responses, form chronic infections. Interestingly, resistant SJL mice have higher ILC2 responses to Hp than susceptible B6 mice (118). This raises the possibility that some helminths may attempt to evade host rejection by modulating ILC2 responses, and in line with this idea McSorley and colleagues recently reported that secretory products from Hp can attenuate allergic airway inflammation by blocking the release of IL-33 from epithelial cells (119). The authors went on to identify one of the proteins responsible, and termed this protein Hp-derived Alarmin Release Inhibitor (HpARI) (120). HpARI selectively binds to IL-33 and traps it in the nucleus, preventing its release during cell apoptosis. This was associated with a reduction in IL-13+ and IL-5+ ILC2s in lungs of mice exposed to *Alternaria*. Hp has also been shown to alter the composition of the intestinal microbiome increasing the availability of the bacterial metabolites, short chain fatty acids (SCFA). The authors went on to demonstrate that Hp-induced production of bacterial SCFAs was able to attenuate house dust mite-induced allergic asthma in mice (121). However, it would also be interesting the determine the impact of SCFAs on the host response to the parasite as butyrate, a SCFA, has recently been shown to directly block IL-13 and IL-5 expression by ILC2s (122), which have been associated with the weep and sweep response.

## ILC2 Responses and Human Helminth Infection

To date, the markers used to identify the various ILC populations in humans remain poorly defined, and very few studies have characterized ILC responses in the context of any human infection (123), let alone helminth infection. Instead most of our knowledge about human ILC2s are derived from studies of inflammatory diseases where ILC2s play a pathological role (*i.e.*, chronic rhinosinusitis, COPD, dermatitis, asthma) (124). Importantly however, these studies have revealed that ILC2s are present in various mucosal tissues of humans including the lungs, the gut and the skin—the same sites where helminths are typically found. In addition, many of the activation pathways described for murine ILC2s, have now been confirmed in humans including the three alarmins (IL-33, IL-25, and TSLP), leukotrienes and tuft cells (38, 69, 125–129).

In terms of helminth infection, Boyd et al. (130) reported an increase of c-kit+ ILCs (similar to ILC2s and ILC3s in mice) in the circulation of people infected with helminth filariae (*Loa loa* or *Wuchereria bancrofti* or *Onchocerca volvulus*). These cells expressed IL-13 and were identified as Lineage (Lin)-/CD45+/cKit+/CD127+, but additionally expressed IL-10 and IL-17 (130). Both *Wuchereria* and *Onchocerca* helminths harbor the bacterial endosymbiont *Wolbachia*, which may bias the host immune response away from a type 2 response and toward a type 17 response and would explain the observed increase in both IL-13 and IL-17 expression. Yet, most of the patients studied were infected only with *Loa loa* (130), which unlike its relatives does not harbor *Wolbachia*. An alternative explanation could therefore be that the increased c-kit ILCs reported in infected patients may be largely LTis rather than ILC2s.

A second study assessed the ILC response in children infected with *Schistosoma haematobium* (131). Younger children exhibited lower numbers of circulating ILC2s, identified as Lin-CD45+CD127+CD294+CD161+, whilst ILC2 numbers in older children were similar between infected and control individuals. Following anthelmintic treatment, the number of ILC2s present in young children was restored to levels apparent in uninfected patients, suggesting that *Schistosoma* may suppress the ILC2 response (131). In *Schistosoma* infection, protective immunity is known to build up over time, and the “older” children had antibody titers indicative of the acquisition of immune protection (131). Determining whether the positive correlation between ILC2 numbers and increasing age simply reflects the slow acquisition of protective type 2 immunity against endemic helminths, or whether these cells actually play a causal role in promoting such protection will be an important question for the future.

Other unanswered questions include: Does the ILC2 response differ following infection with different families or species of helminths? Are there fundamental differences in the ability of young children or adults to generate ILC2s? Do ILC2 numbers correlate with disease phenotypes including resistant (non-infected but exposed), susceptible (clinically symptomatic, infected), or controller (clinically asymptomatic, infected) individuals? These questions may have fundamental importance for the design of successful vaccines against these widespread, and often debilitating, parasites.

## Conclusions and Perspective

Our understanding and knowledge of ILC2s has expanded tremendously over the recent decade, yet much remains to be determined. Whilst the majority of existing research related to human ILC2s have focused on the “first-world” diseases related to allergic inflammation, we would argue that attention should also be given to the role of these cells during human helminth infection given the clear need for improved control of these parasites amongst developing societies. In addition to this, ongoing studies of ILC2s in the context of host-helminth interactions—either in mice or humans—are highly likely to continue to shed light on the activation, regulation, and function of these cells. In terms of allergic disease, mining helminths for molecules that suppress ILC2 responses could represent a promising avenue for the identification of novel therapeutics. Lastly, at a time when we are just beginning to understand the full importance of ILC2s in anti-helminth immune responses, Maizels and colleagues recently described the existence of another, as yet undefined but very rare innate immune cell, that is important for the expulsion of Hp (132). This report highlights that our understanding of type 2 immune responses and of host-helminth interactions is continually evolving. Although studies performed over the last decade led to the discovery of ILC2s, it is likely that continued efforts in this area will reveal many interesting, and perhaps even surprising, facets of the type 2 immune response.

## Author Contributions

All authors listed have made a substantial, direct and intellectual contribution to the work, and approved it for publication.

### Conflict of Interest Statement

The authors declare that the research was conducted in the absence of any commercial or financial relationships that could be construed as a potential conflict of interest.
